# CDC27-ODC1 Axis Promotes Metastasis, Accelerates Ferroptosis and Predicts Poor Prognosis in Neuroblastoma

**DOI:** 10.3389/fonc.2022.774458

**Published:** 2022-02-15

**Authors:** Lin Qiu, Rui Zhou, Ziyan Luo, Jiangxue Wu, Hua Jiang

**Affiliations:** ^1^ Department of Hematology/Oncology, Guangzhou Women and Children’s Medical Center, Guangzhou Medical University, Guangzhou, China; ^2^ Department of General Surgery, The Third Affiliated Hospital of Southern Medical University, Southern Medical University, Guangzhou, China; ^3^ Department of Hepatobiliary Surgery, Sun Yat-sen Memorial Hospital, Sun Yat-sen University, Guangzhou, China; ^4^ State Key Laboratory of Oncology in South China, Collaborative Innovation Center for Cancer Medicine, Sun Yat-sen University Cancer Center, Guangzhou, China

**Keywords:** CDC27, ODC1, neuroblastoma, metastasis, ferroptosis

## Abstract

Neuroblastoma (NB) is a devastating malignancy threatening children’s health, and amplification of MYCN is associated with treatment failure and a poor outcome. Here, we aimed to demonstrate the role of cell division cycle 27 (CDC27), an important core subunit of the anaphase-promoting complex, and its clinical significance in NB patients. In functional assays, we illustrated that CDC27 promoted the cell growth, metastasis and sphere-formation ability of NB cells both *in vitro* and *in vivo*. To further understand the potential mechanism, SK-N-SH cells were transfected with CDC27 siRNA, and RNA-sequencing was performed. The results revealed that downregulation of CDC27 led to markedly reduced expression of ODC1, which is a well-established direct target of MYCN. Subsequently, we further illustrated that suppression of ODC1 significantly attenuated the promotion effect of CDC27 on the proliferation, metastasis, and sphere-formation ability of NB cells, hinting that CDC27 exerted its biological behavior in NB at least partly in an ODC1-dependent manner. In addition, CDC27 rendered cells more vulnerable to ferroptosis, while knockdown of ODC1 markedly reversed the pro-ferroptotic effect of CDC27. Collectively, our data is the first to report that the CDC27/ODC1 axis promotes tumorigenesis and acts as a positive regulator of ferroptosis in NB, highlighting that CDC27 may represent a novel therapeutic strategy and prognostic biomarker in neuroblastoma.

## Introduction

Neuroblastoma (NB) is a peripheral neural system tumor that derives from the primitive neural crest. High-risk NB is always characterized by amplification of MYCN, which is associated with treatment failure and indicates an extremely poor outcome (1–4). High-risk NB patients suffer from therapeutic resistance, refractory or relapsed disease, and distant metastasis remains the main cause of death (5, 6). MYCN always exerts its protumorigenic effects by transcriptional regulation of target oncogenes. Hence, considerable efforts have been concentrated on the critical target genes and pathways of MYCN, such as ODC1, TERT, ID2, and polyamine pathways (7–12).

Cell division cycle 27 (CDC27) is an important core subunit of anaphase-promoting complex/cyclosome (APC/C) (13, 14), which has been found to be mutated in several malignant tumors, implying that CDC27 may be dysregulated in various types of tumors (15, 16). In our previous studies, we have reported that CDC27 promoted tumor progression and served as an independent prognostic factor in colorectal cancer (17, 18). However, there is also evidence indicating that CDC27 may act as a tumor suppressor in breast cancer (19). Therefore, the function of CDC27 may be cell- and tissue-specific in cancer. Thus far, the role of CDC27 in NB still remains unknown.

Polyamines participate in many cellular processes governed by MYC genes, which have been identified as a hallmark of neuroblastomas with MYCN amplification (20). Ornithine decarboxylase 1 (ODC1), is a key rate-limiting enzyme in polyamine synthesis. Besides, ODC1 is also a well-characterized target of MYCN, which has been reported to be decisive in mediating the effects of MYCN in NB (6, 10).

The therapeutic effect of inducing cell apoptosis with principal anticancer drugs is limited owing to the acquired or intrinsic resistance of cancer cells to apoptosis (21, 22). Thus searching for alternative nonapoptotic cell death pathways may provide new insights for eliminating cancer cells (23). Ferroptosis is a new form of iron-dependent cell death, characterized by the accumulation of reactive oxygen species (ROS) and lipid peroxidation (22). Ferroptosis has a dual role in tumor promotion and suppression, which depends on the release of damage-associated molecular patterns and the activation of the immune response triggered by ferroptosis damage within the tumor microenvironment (24). In recent years, emerging evidence illustrated that MYCN increased iron influx in NB cells, leading to a marked vulnerability to ferroptosis inducers of NB cells, which suggests that inducing ferroptosis in NB cells may be a potential strategy in treating high-risk NB (25, 26). Studies have elucidated that the rate-limiting enzyme in polyamine catabolism (27), is involved in the regulation of ferroptosis, however, the roles of the enzymes involved in polyamine catabolism in ferroptosis remain unknown in NB.

In this study, we first discovered the function of the CDC27/ODC1 axis in NB, and proposed that CDC27 may serve as a novel regulator of ferroptosis in an ODC1-dependent manner, providing evidence that the CDC27-ODC1 axis may serve as a promising candidate therapeutic target for NB.

## Materials and Methods

### Cell Culture and Transfection

The cell lines SK-N-SH, and SH-SY5Y were purchased from Procell Life Science & Technology (CL-0214, CL-0208, Wuhan, China). SK-N-SH, SH-SY5Y, SK-N-BE(2) cells were cultured in MEM (C11095500BT, Gibco, NY, USA) containing 10% fetal bovine serum and an appropriate concentration of penicillin-streptomycin antibiotics (15140-122, Gibco, NY, USA). Cell line authentication was performed using short tandem repeat DNA profiling, and mycoplasma detection was regularly performed using a PCR-based method. All cell lines were cultured in a humidified atmosphere with 5% CO2 at 37°C.

Plasmids carrying CDC27 were constructed previously (17). Premixed siRNA was purchased from Santa Cruz (siCDC27:sc-77362, siNC: sc-37007). Growing cells were added to six-well plates and then transfected with plasmid or siRNA using Lipofectamine 2000 transfection reagent (Invitrogen) following the manufacturer’s instructions.

### Gene Expression Profile Datasets

The gene expression array GSE45547 was downloaded from the Gene Expression Omnibus (GEO, http://www.ncbi.nlm.nih.gov/geo). Related clinical data were collected from the R2 database and a Kaplan–Meier analysis was performed using a website tool (https://hgserver1.amc.nl/cgi-bin/r2/main.cgi). The cohort contained 649 patients and a total of 476 subjects had survival data. The average expression level of CDC27 (also known as UKv4_A_23_P66777 in the R2 dataset) was used as the cutoff value, and patients were divided into two groups (low/high expression) according to the average CDC27 expression, and a raw p value was obtained.

### Patient Tissue Specimens and Immunohistochemistry (IHC)

Patients who did not have follow-up information were excluded from this study. Patients who had a single primary lesion and no neoadjuvant chemotherapy were included. The study was approved by the Ethics Committee of Guangzhou Women and Children’s Medical Center in 2018 (approval number 2018030355), and all patients provided informed consent. A total of 121 paraffin-embedded tissue samples, including neuroblastoma (NB, n=66), ganglioneuroblastoma (GNB, n=30), and ganglioneuroma (GN, n=25), were collected from the Guangzhou Women and Children’s Medical Center. CDC27 antibody (10918-1-AP, Proteintech, China) was purchased from Proteintech. Immunohistochemistry (IHC) staining was performed as below in brief. All sections were deparaffinized in xylene, rehydrated in ethanol, and blocked in methanol with 0.3% H^2^O^2^. The tissue antigens were then retrieved in citrate buffer (pH 6.0). Then, the sections were treated with primary antibodies, and incubated overnight at 4°C in a humidified chamber. Next, the slides were incubated with a secondary antibody for 30 min and stained with diaminobenzidine (DAB) tetrahydrochloride. Finally, the sections were counterstained with hematoxylin, dehydrated, and mounted. Three independent observers who were not informed of the clinical data of the patients evaluated the staining intensity. The expression intensity was classified as negative = 0, weak = 1, moderate = 2, or strong = 3. The final H score was calculated based on multiplying the intensity score by the percentage of the staining area as described previously (17). A receiver operating characteristic (ROC) curve analysis was applied to determine a cutoff value for CDC27 low expression and high expression. A total of 70 patients (NB, n=44; GNB, n=26) with follow-up information were included in the Kaplan–Meier survival analysis. The associated clinical information of the patients is summarized in **Supplementary Table S1**.

### Stable Cell Line Construction

To construct stable cell lines with CDC27 knockdown or overexpression, lentiviral vectors carrying CDC27 shRNA or cDNA were produced by GeneChem Co., Ltd. (Shanghai, China), and used to infect SK-N-SH and SH-SY5Y cells. After 48 h of infection, stably infected cells were selected with puromycin (2 μg/ml) for two weeks. Real-time PCR and western blotting were used to evaluate the expression of CDC27.

### RNA Extraction and Quantitative Reverse Transcription PCR

Total RNA was isolated using TRIzol reagent (15596026, Invitrogen, CA, USA), and conversion to cDNA was performed using the GoScript Reverse Transcription System (A5003, Promega, WI, USA). The mRNA expression levels of relevant target genes were quantified by real-time PCR using a SYBR Green PCR Kit (4309155, Thermo Fisher Scientific, MA, USA) and normalized to β-actin. The primers used are listed in **Supplementary Table S2**. PCR was carried out with 40 cycles of a 2-step reaction (denaturing at 95°C for 10 seconds, and annealing and elongation at 60°C for 30 seconds).

### RNA-Sequencing Analysis

Total RNA was extracted from SK-N-SH cells transfected with siCDC27 or siNC. RNA-sequencing analysis was performed by Guangzhou RiboBio Co., Ltd. The RNA-seq data have been deposited in the ArrayExpress database at EMBL-EBI (www.ebi.ac.uk/arrayexpress) under accession number E-MTAB-11257.

### Western Blotting

The detailed procedure was described previously (28). In brief, cell lysates were separated by SDS–PAGE and transferred to polyvinylidene difluoride membranes (Roche, Basel, Switzerland). Next, after blocking in 5% skimmed milk, membranes were sequentially incubated with corresponding primary antibodies overnight at 4°C, followed by incubation with peroxidase-coupled secondary antibodies. Finally, the blots were visualized using an electrochemiluminescence system. The primary antibodies used were as follows: CDC27 (1:1000, 10918-1-AP, Proteintech, China), ODC1 (1:1000, 28728-1-AP, Proteintech, China), and GAPDH (1:1000, 60004-1-Ig, Proteintech, China).

### Cell Viability Assays

MTT and colony formation assays were applied to evaluate cell viability. For MTT assays (M1020, Solarbio, Beijing, China), the indicated cells were seeded in 96-well plates, and the absorbance of each sample was measured at 490 nm. For colony formation assays, the indicated cells were plated into six-well plates and supplemented with complete medium containing 10% FBS. After incubating at 37°C for 10–14 days, the cells were washed, and visible colonies were fixed and stained with methanol and 0.5% crystal violet. The average of 3 repeated experiments was calculated.

### Cell Migration and Invasion Assay

In brief, the indicated cells resuspended in 200 µL free medium were added to the upper compartment of chambers coated with or without Matrigel for invasion and migration assays, respectively. The bottom chamber was filled with specified medium with 10% FBS. Cells that migrated to the lower surface of the chamber were fixed, stained at the indicated times, and counted at 100× magnification in five randomly selected fields. Experiments were independently repeated three times.

### Wound Healing Assays

Briefly, the indicated cell lines were seeded in 6-well plates and incubated for 24 h. Subsequently, 200 μL pipette tips were used to scratch the cell monolayers, and the cells were gently washed twice using PBS. Cells were cultured in standard FBS-free medium. Scratch wound formation images were captured at the indicated time point with a magnification of 100× using an inverted microscope.

### Sphere-Formation Assay

Cells were suspended in serum-free DMEM/F12 (11320033, Gibco, NY, USA) supplemented with penicillin–streptomycin antibiotics, 20 ng/ml human epidermal growth factor (hEGF, 8916SF, Cell Signaling Technology, MA, USA), 20 ng/ml human fibroblast growth factor (hFGF, 33016015, Thermo Fisher, MA, USA), and 2% B27 Supplement (17504044, Gibco, NY, USA) without vitamin A. Subsequently, the indicated cells were cultured in ultralow attachment 24-well plates (3473, Corning, NY, USA) at a density of 1000 cells/well, and the size and number of cell spheres were evaluated *via* microscopy after 10 days.

### Water-Soluble Tetrazolium (WST) Assays

A Cell Counting Kit-8 assay was applied to evaluate cell viability according to the manufacturer’s instructions (HY-K0301, MedChem Express, NJ, USA) for WST-8. Viability values were obtained by a microplate absorbance reader at 450 nm. The cell growth inhibition rate was calculated following the manufacturer’s instructions.

### Chemicals

In the ferroptosis-related assays, commercial ferroptosis inducers RSL3 (S8155), and BSO (S9728), ferroptosis inhibitor ferrostatin-1 (S7243), caspase inhibitor Z-VAD-FMK (S7023) and necroptosis inhibitor necrostatin-1 (S8037) were purchased from Selleck (Shanghai, China).

### Lipid Peroxidation Measurement

The levels of malondialdehyde (MDA) were analyzed to detect the well-known lipid peroxidation marker of ferroptosis, using a Lipid Peroxidation Assay Kit (ab243377, Abcam, Cambridge, USA) according to the protocols listed in the official instructions.

### Reduced Glutathione Assay

The Reduced Glutathione Assay Reagent Kit (BC1175, Solarbio, Beijing, China) was applied to detect the extent of decreased glutathione synthesis according to the manufacturer’s instructions.

### Iron Assays

For the iron assays, we used an Iron Assay Kit (MAK025-1KT, Sigma Aldrich, MO, USA) to measure the level of iron in the indicated cells according to the manufacturer’s instructions.

### Animal Experiments

All animal experiments were approved by the Institutional Animal Care and Use Committee of Guangzhou Women and Children’s Medical Center in 2018 (ethical approval number 2018-604). Male BALB/c nude mice (4-5 weeks old, 15-18 g) were purchased from GemPharmatech Co., Ltd. (Nanjing, China) and were randomly assigned to four groups (n=8 per group).

For tumor formation assays, a total of 2×10^6^ cells in 100 μL of PBS were subcutaneously injected into the right flanks of the nude mice. The tumor sizes were measured at the indicated time points. After 28 days, mice were euthanized, and the primary tumors were collected and weighed. The tumor volumes were calculated using the following formula: V = (width^2^× length)/2 (5).

For lung metastasis models, a total of 1×10^6^ indicated cells resuspended in 100 μL of PBS were injected into the tail veins. Mice were sacrificed at 10 weeks after injection, and the lungs were harvested, weighed and stained with hematoxylin and eosin for histological analysis. The number of metastatic nodules was counted under a microscope.

### Statistical Analysis

All statistical analyses were performed using SPSS version 16.0 software. Normally distributed data are presented as the means ± SD. Differences in the quantitative variables between groups were analyzed by Student’s t test. Survival curves were plotted by the Kaplan–Meier method and were compared using the log-rank test. Statistical significance was defined as *P<*0.05. Each experiment was performed independently at least three times.

## Results

### Elevated Expression of CDC27 Predicted a Poor Outcome in NB

To demonstrate the role of CDC27 in NB metastasis, we first detected CDC27 expression in primary NB and paired metastatic tumor tissues. The results indicated that the mRNA expression of CDC27 was upregulated in the paired metastatic tumor tissues compared to the primary tumor tissues (**Figure S1A**). We also examined CDC27 expression in several NB cell lines. The results indicated that CDC27 was frequently upregulated in NB cell lines compared with normal dorsal root ganglion cells (**Figure S1B**).

**Figure 1:**
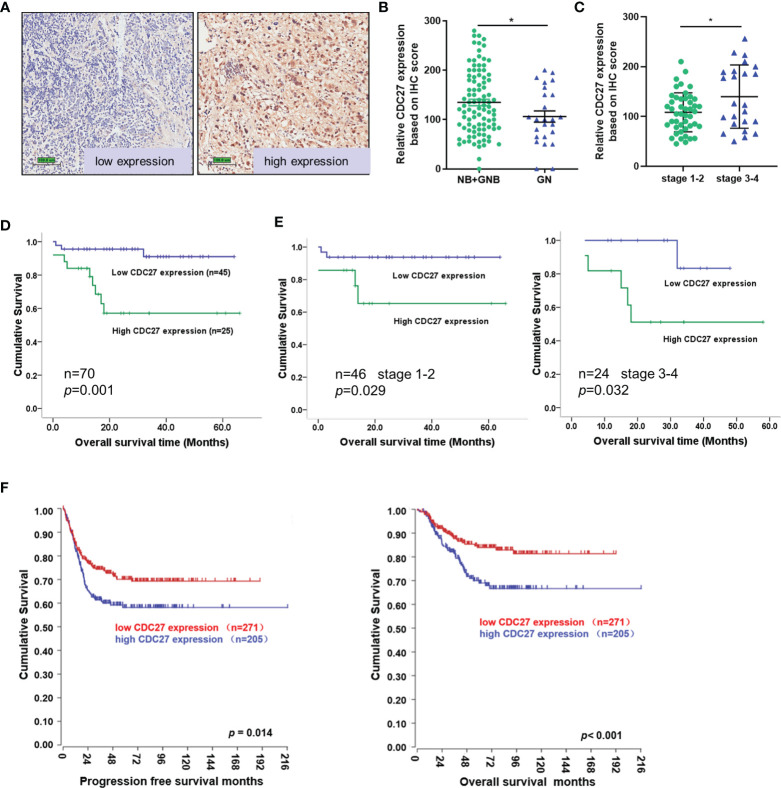
CDC27 predicted poor prognosis of NB patients. **(A–C)** Representative images of CDC27 expression detected by IHC in NB tissues; each point in the graph represents the CDC27 expression H-score of an individual patient tumor. *p < 0.05 based on Student’s t test. **(D, E)** Kaplan–Meier survival analysis of OS according to CDC27 expression evaluated by IHC in 70 collected NB patient tissues. **(F)** Kaplan–Meier survival analysis of the association between CDC27 expression and PFS or OS according to the Gene Expression Omnibus (GEO) data.

To assess the clinical significance of CDC27 in NB, we performed immunochemistry using tissue sections from 121 NB patients. The results revealed that the expression of CDC27 is related to the malignancy degree of NB, because CDC27 expression was not only markedly increased in NB and GNB tissues compared with GN tissues (NB+GNB n=96, GN n=25; **Figures 1A, B**), but also higher in stage 3-4 NB patients than in stage 1-2 NB patients (**Figure 1C**). Additionally, we analyzed the association between CDC27 expression and the survival time of 70 NB patients with clinical follow-up information. A Kaplan**–**Meier survival analysis demonstrated that patients with high expression of CDC27 had a significantly shorter overall survival (OS) time (**Figures 1D, E**).

In addition, we obtained an expression array from a public database to further validate the prognostic value of CDC27. A total of 476 subjects from GSE45547 were selected. Consistent with our results described above, the Kaplan**–**Meier survival analysis indicated that high CDC27 expression resulted in worse progression-free survival (PFS) and OS rates (**Figure 1F**). Collectively, the results above indicated that CDC27 predicted a poor prognosis in NB.

### CDC27 Promoted the Cell Growth and Mobility of Neuroblastoma Cells

**Figure 2:**
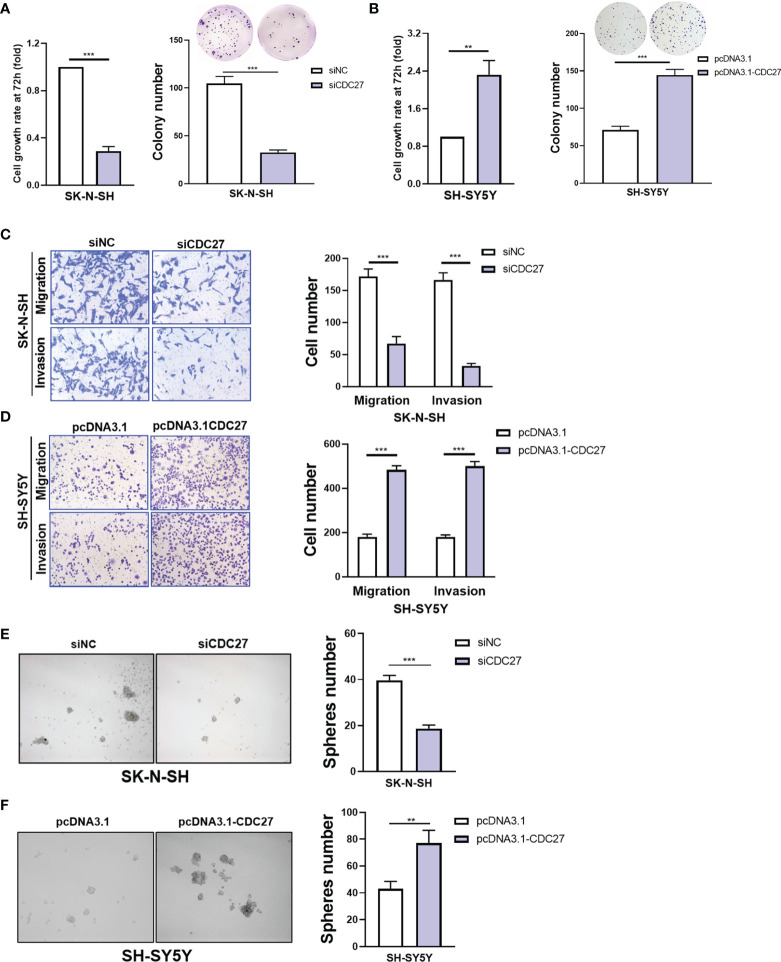
CDC27 promoted cell growth and mobility in NB cells. **(A, B)** Cell viability and colony-formation ability of the indicated cells with CDC27 downregulation or upregulation were evaluated by MTT and colony formation assays. **(C, D)** Representative images of Transwell assays show the migration and invasion ability of the indicated cells with CDC27 overexpression or knockdown. Cell numbers were quantified. **(E, F)** Representative images show the sphere-formation capability of the indicated cells with CDC27 overexpression or suppression. ***p < 0.001, **p < 0.01 based on Student’s t test. These experiments were repeated at least three times. Error bars, mean ± SD.

Next, we aimed to explore the role of CDC27 in NB by performing cell function assays. In view of the endogenous expression level of CDC27 in the NB cell lines above, SK-N-SH and SH-SY5Y cells were selected to transiently knock down or overexpress CDC27 using siRNA (siCDC27, siNC as a negative control) and CDC27-carrying plasmids (pcDNA3.1-CDC27, pcDNA3.1 as a negative control), respectively. The transfection efficiency was validated by qRT**–**PCR and western blot (**Figures S2A, B**). The proliferation capability of NB cells was determined by MTT and colony formation assays. We observed that suppression of CDC27 led to reduced cell proliferation in SK-N-SH cells, while overexpression of CDC27 markedly accelerated cell growth in SH-SY5Y cells (**Figures 2A, B**). Transwell assays revealed that downregulation of CDC27 suppressed cell migration and invasion in SK-N-SH cells, and elevated expression of CDC27 enhanced the migration and invasion capacities in SH-SY5Y cells (**Figures 2C, D**). In addition, similar results were obtained from wound healing assays, in which CDC27 accelerated the wound healing abilities of both SK-N-SH and SH-SY5Y cells (**Figure S3**), which reinforced the conclusion that CDC27 promoted metastasis in neuroblastoma.

We also performed sphere-formation assays. As shown in **Figures 2E, F**, CDC27 positively regulated the sphere-formation capacity of SK-N-SH and SH-SY5Y cells. Collectively, our results illustrated that CDC27 can promote proliferation, metastasis, and sphere-formation ability in NB cell lines.

### Identification of ODC1 as a Potential Target of CDC27

**Figure 3:**
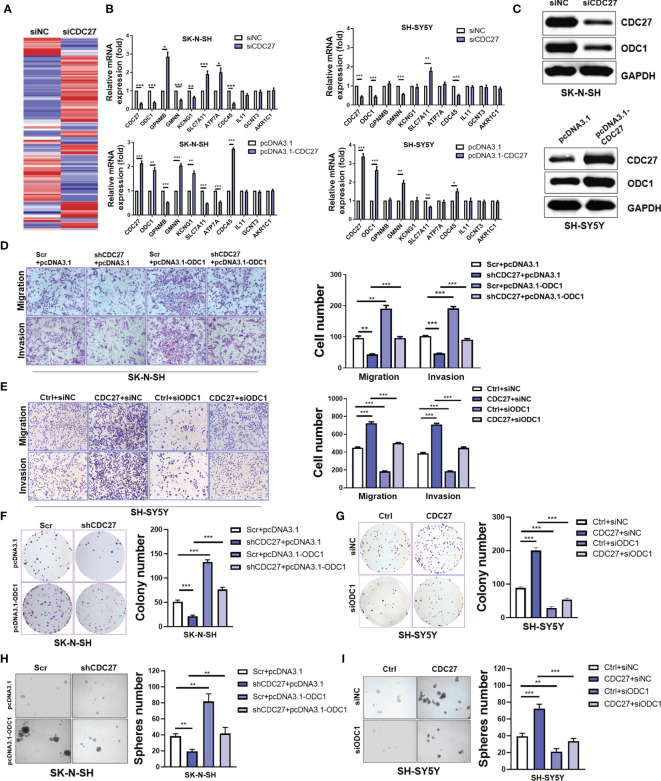
CDC27 promoted proliferation and metastasis by ODC1 activation. **(A)** Heatmap for differentially expressed genes from RNA-sequencing results. **(B)** Relative mRNA expression level of selected genes normalized to β-actin level in SK-N-SH and SH-SY5Y cells with CDC27 transiently knocked down or overexpressed by real time RT–PCR. **(C)** Validation of ODC1 expression by western blot. **(D–I)** ODC1 was transiently overexpressed or suppressed in the indicated stable cell lines. Representative images of colony formation, Transwell, and sphere-formation rescue assays are shown, and quantifications are presented. The means ± SD of triplicate samples are shown. ***p < 0.001, **p < 0.01, *p < 0.05 based on Student’s t test.

To gain further insight into the mechanism by which CDC27 exerts its biological behavior in NB, we sought to identify the downstream molecule of CDC27. SK-N-SH cells were transiently transfected with siCDC27 or siNC for RNA sequencing, and a heatmap of differentially expressed genes is shown in **Figure 3A** and **Supplementary Information 1**. We selected ten significantly differentially expressed genes with relatively high endogenous expression, and validated the results in two NB cell lines. SK-N-SH and SH-SY5Y cells were both transfected with siCDC27 or pcDNA3.1-CDC27. As a result, four genes (ODC1, GMNN, SLC7A11, CDC45) were confirmed to be significantly regulated by CDC27 in both cell lines (**Figure 3B**).

Based on the above findings, we focused on ODC1, the first rate-limiting enzyme in the polyamine synthesis pathway. ODC1 is a critical target of MYCN, and inhibition of ODC1 could impair the development of MYCN-amplified NB (29), implying that elucidating the aberrant activation of ODC1 will provide vital evidence for NB treatment. Given the crucial role of ODC1 in NB, we focused on whether ODC1 was indeed regulated by CDC27 in NB cells. As shown in **Figure 3C**, western blot analysis revealed that ODC1 was activated by CDC27 in both SK-N-SH and SH-SY5Y cells.

Next, stable cell lines with CDC27 knockdown and corresponding control cell lines were established using SK-N-SH cell lines, named shCDC27 and Scr, respectively. CDC27-overexpressing and corresponding control stable cell lines were constructed using SH-SY5Y, and named Ctrl and CDC27. These stable cell lines were transfected with plasmid-carrying ODC1 (pcDNA3.1-ODC1, pcDNA3.1 as a negative control) or ODC1 siRNA (siODC1, siNC as a negative control). The results revealed that overexpression of ODC1 abrogated the inhibitory effects on proliferation, metastasis, and sphere-formation ability caused by knockdown of CDC27 (**Figures 3D, F, H** and **Figure S3C**). Similarly, knockdown of ODC1 markedly reversed the promotion effects on cell growth, mobility and sphere-formation caused by CDC27 (**Figures 3E, G, I** and **Figure S3D**). Similar results were also observed in SK-N-BE(2) cells (**Figure S4**). Thus, we hold that CDC27 exerts its protumorigenic effects in NBs in an ODC1-mediated manner.

### CDC27 Promoted the Growth and Metastasis of NB Cells *In Vivo*

**Figure 4:**
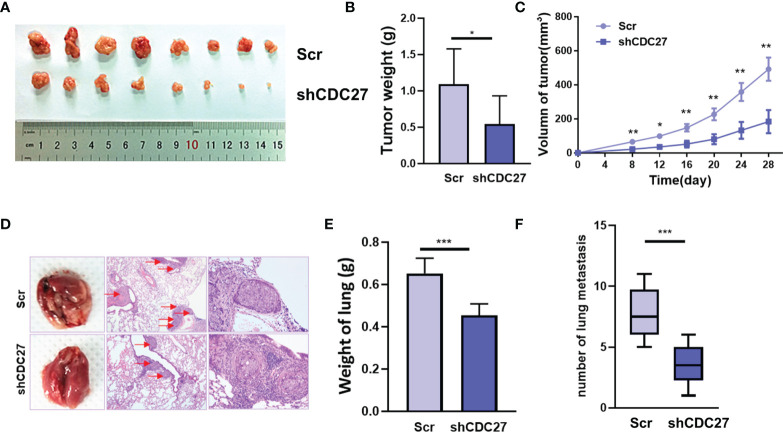
CDC27 promoted the proliferation and metastasis of NB cells *in vivo*. **(A)** Xenograft tumors were collected 28 days after cell injection. **(B, C)** The weights of the tumors were analyzed, and data points are presented as the means ± SD for tumor volumes. **(D)** Representative images of metastatic lung nodules of HE-stained sections are shown, and the metastatic nodules are highlighted by red arrows. **(E, F)** The weight of the lungs and the number of lung metastatic nodules were analyzed. ***p < 0.001, **p < 0.01, *p < 0.05 based on Student’s t test.

To further confirm the *in vitro* results above, we established xenograft models, including a subcutaneous xenograft model, and lung metastasis models. We observed that the weight and volume of the xenografts were both significantly reduced in the group treated with shCDC27 cells compared with the control group (**Figures 4A**–**C**). Furthermore, the results from lung metastasis models showed that downregulation of CDC27 caused dramatic reduction in lung weight and the number of metastatic nodules compared with the control group (**Figures 4D–F**), all of which were in accord with the results of *in vitro* assays.

### CDC27 Contributed to Ferroptosis in NB Cells

**Figure 5:**
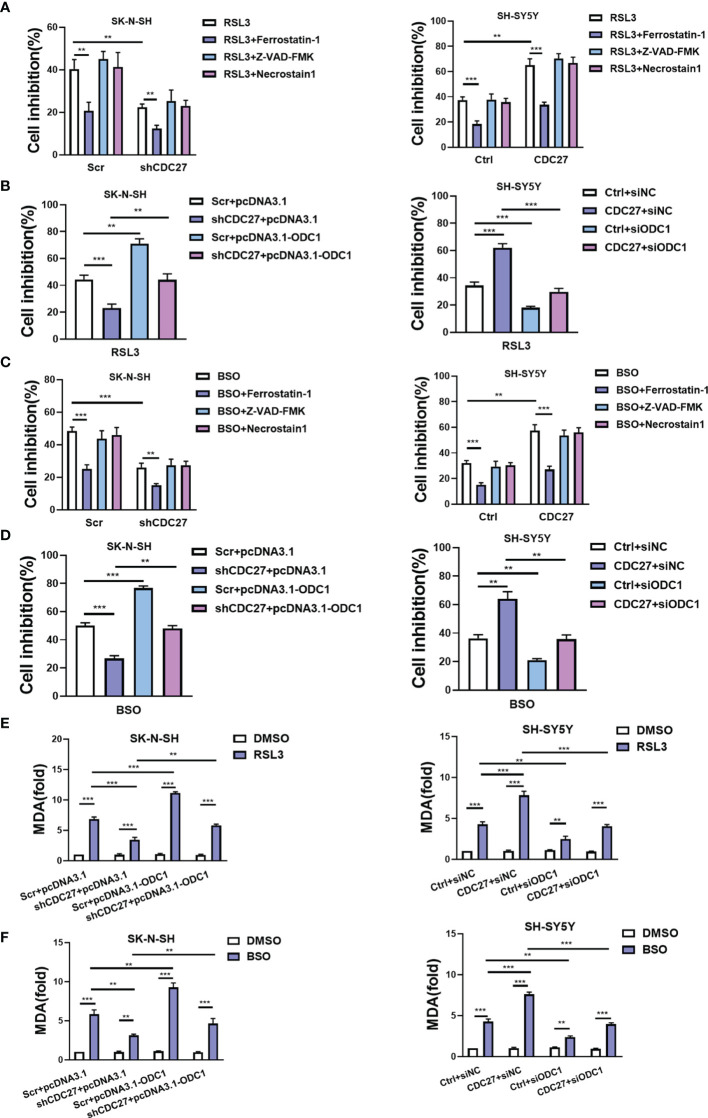
CDC27 contributes to ferroptosis of NB cells *via* regulation of ODC1. **(A, C)** The indicated cells were treated with RSL3 (ferroptosis inducer, 1 μM) or BSO (ferroptosis inducer, 5 μM), ferrostatin-1 (ferroptosis inhibitor, 1 μM), Z-VAD-FMK (caspase inhibitor, 10 μM) or necrostatin-1 (necroptosis inhibitor, 10 μM) for 24 h, and the WST assay results are shown. **(B, D)** The indicated cells with transient overexpression or suppression of ODC1 were treated with RSL3 or BSO for 24 h. WST assays were performed. **(E, F)** MDA content was detected in the indicated cell lines. Means ± SD of triplicate samples are shown. **p < 0.01, ***p < 0.01 based on Student’s t test.

Ferroptosis is identified as an iron-dependent nonapoptotic mode of cell death, triggered by reduced cystine uptake, decreased glutathione synthesis, and the accumulation of lipid peroxidation. Based on the fact that ODC1 is a key rate-limiting enzyme in polyamine synthesis, and that polyamine metabolism is involved in ferroptosis regulation (27), we proposed whether the CDC27-ODC1 axis participates in ferroptosis in NB. In the experiments below, the indicated cells were treated with RSL3, BSO (ferroptosis inducer), ferrostatin-1 (ferroptosis inhibitor), Z-VAD-FMK (caspase inhibitor) or necrostatin-1 (necroptosis inhibitor) for 24 h. Notably, the results from WST assays revealed that the growth rates of both SK-N-SH and SH-SY5Y cells were substantially inhibited in the presence of RSL3, while the addition of ferrostatin-1 significantly weakened the growth inhibitory effect of RSL3, and Z-VAD-FMK and necrostatin-1 treatment caused no significant difference (**Figure 5A**). Similar to ferrostatin, downregulation of CDC27 also impeded the inhibitory effect of RSL3 on cell growth in SK-N-SH cells, while upregulation of CDC27 made SH-SY5Y cells more sensitive to RSL3-induced ferroptosis (**Figure 5A**), hinting that CDC27 accelerated RSL3-induced ferroptosis in neuroblastoma cells.

In addition, exogenous expression of ODC1 markedly reversed the antiferroptotic effect caused by CDC27 downregulation in SK-N-SH cells. Likewise, suppression of ODC1 abrogated the effect of CDC27 on promoting ferroptosis in SH-SY5Y cells (**Figure 5B**). We applied another ferroptosis inducer BSO to further validate the results above, and similar results were observed in NB cells with BSO-induced ferroptosis (**Figures 5C, D** and **Figures S6A, B**).

Next, glutathione assays, iron assays, and lipid peroxidation assays were performed to detect the levels of GSH, iron, and MDA (a well-known lipid peroxidation marker) in the indicated cells treated with RSL3 or BSO. Results revealed that BSO treatment caused decreased GSH levels, and knockdown of CDC27 alleviated the BSO induced GSH reduction in NB cells, while overexpression of ODC1 significantly reversed this effect (**Figures S5A**, **S6C**). Consistent results were obtained in CDC27-overexpressing SH-SY5Y stable cell lines in the glutathione assays (**Figure S5B**). In the iron assays, knockdown of CDC27 reduced the accumulation of intracellular iron, while overexpression of ODC1 markedly reversed the effect caused by knockdown of CDC27 in SK-N-SH cells (**Figure S5C**). Likewise, upregulation of CDC27 led to increased intracellular iron levels, and suppression of ODC1 significantly abrogated the effect of CDC27 in SH-SY5Y cells (**Figure S5D**). Additionally, RSL3 treatment led to the accumulation of MDA in the indicated treated cells and downregulation of CDC27 caused significantly reduced MDA content compared with the control group in NB cells, while the effect could be partially reversed by the exogenous expression of ODC1(**Figure 5E**). Consistent results were obtained in the SH-SY5Y stable cell lines (**Figure 5E**). Moreover, a similar phenomenon was observed in the indicated cells treated with BSO (**Figure 5F**).

We further analyzed several key ferroptosis-associated markers to explore the mechanisms by which the CDC27/ODC1 axis regulates ferroptosis. Fortunately, the western blot results showed that CDC27 inhibited the expression of SLC7A11 (**Figures S7A, B**), a well-known key negative regulator of ferroptosis (20, 21), which was also validated by our RNA-sequencing results as shown in **Figure 3B**. Thus, the role of the CDC27/ODC1 axis in promoting ferroptosis was further demonstrated by ferroptosis markers. Therefore, we concluded that CDC27 rendered NB cells more vulnerable to ferroptosis conferred by ODC1 upregulation.

## Discussion

Neuroblastoma (NB) is the most common extracranial solid malignancy in children, and tumor metastasis and recurrence play substantial roles in tumor-caused death. Therefore, understanding the molecular and genetic properties related to NB is necessary for developing promising treatments (30–32). In this study, we identified that CDC27 was associated with poor patient survival, and promoted proliferation and metastasis both *in vitro* and *in vivo*, implying that CDC27 may be a promising druggable target in NB.

Patients with high-risk NB are often characterized by amplifications of MYCN, which predicts an unfavorable prognosis in NB (29). Downregulation of MYCN could suppress tumorigenesis in NB, however, there are technical challenges in directly targeting MYCN clinically (10, 33). Thus, drug development antagonizing target genes or pathways of MYCN has attracted broad attention due to its considerable clinical application value. ODC1, a well-characterized direct target of MYCN, may provide such a target (8, 10, 34–36).

ODC1 is recognized as a critical determinant of MYCN in oncogenesis, for that ODC1 could substitute MYCN to transform cells both *in vitro* and *in vivo*, and the effects of MYCN on tumor initiation and progression can be attenuated through repression of ODC1 (10, 37–39). DFMO is a specific inhibitor of ODC1, however, either DFMO alone or in combination with other chemotherapies has generally been found to have unsatisfactory effects (7, 40). Hence, exploring new mechanisms of ODC1 activation is attractive. In our study, we discovered that CDC27 activated the expression of ODC1 (**Figure 3B**), and promoted tumorigenesis of NB cells in an ODC1-mediated manner (**Figures 3D–I**).

Accelerating programmed cell death is an important chemotherapy approach in cancer therapy. However, some of the key hallmarks in the apoptotic pathway are absent in advanced NB (41, 42); thus, searching for new forms of cell death may open promising therapeutic avenues for NB. Ferroptosis is a newly identified cell death mode, and inducing ferroptosis is of great value because tumor cells need more iron than nonmalignant cells; thus, ferroptosis could kill tumor cells while protecting normal cells from being attacked (43, 44). A few studies have reported that inducing ferroptosis contributes to antitumor effects in NB (25, 45, 46), however, reports concerning ferroptosis in NB remain limited, and the related molecular mechanism is largely unknown.

From the RNA-sequencing data, we noticed that CDC27 inhibited the expression of SLC7A11, which is a unit of the glutamate-cystine antiporter Xc^-^, resulting in increased cystine uptake, and then suppresses lipid oxidation and ferroptosis. In addition, there is evidence indicating that ODC1 participates in the ROS stress response and ferroptosis (47), both of which prompted us to propose that CDC27 may take part in the ferroptosis process. Fortunately, we indeed demonstrated that CDC27 made cells more sensitive to ferroptosis, and downregulation of ODC1 rendered cells refractory to ferroptosis (**Figure 5** and **Figures S5** **–** **S7**).

However, there still exists issues that deserves to be discussed. Ferroptosis has been reported to has a dual role in both tumor promotion and suppression. Although ferroptosis inducer reagents can induce ferroptosis and suppress tumor growth, ferroptosis damage can also trigger inflammation associated immunosuppression in the tumor microenvironment, which leads to tumor growth (24). Thus, whether CDC27 is involved in inflammation-associated immunosuppression, and thereby promotes tumorigenesis of NB cells due to ferroptosis deserves further evaluation in future studies.

Taken together, our study first demonstrated that CDC27 promoted tumorigenesis, metastasis, predicted poor prognosis and was involved in the regulation of ferroptosis in an ODC1-mediated manner in NB. CDC27 may provide new access for therapeutic intervention in NB.

## Data Availability Statement

The original contributions presented in the study are included in the article/**Supplementary Material**. Further inquiries can be directed to the corresponding authors.

## Ethics Statement

The studies involving human participants were reviewed and approved by Ethics Committee of Guangzhou Women and Children’s Medical Center. Written informed consent to participate in this study was provided by the participants’ legal guardian/next of kin. The animal study was reviewed and approved by Institutional Animal Care and Use Committee of Guangzhou Women and Children’s Medical Center.

## Author Contributions

LQ, JW, and HJ designed the experiments. RZ and ZL conducted the experiments. RZ collected the clinical data and analyzed the experimental data. LQ wrote the manuscript. All authors contributed to the article and approved the submitted version.

## Funding

This work was supported by the National Natural Science Foundation of China (No. 81802488).

## Conflict of Interest

The authors declare that the research was conducted in the absence of any commercial or financial relationships that could be construed as a potential conflict of interest.

## Publisher’s Note

All claims expressed in this article are solely those of the authors and do not necessarily represent those of their affiliated organizations, or those of the publisher, the editors and the reviewers. Any product that may be evaluated in this article, or claim that may be made by its manufacturer, is not guaranteed or endorsed by the publisher.

## Supplementary Material

Supplementary Figure 1
**(A)** Analysis of CDC27 mRNA expression in 12 pairs of primary and metastatic patient tumor tissues. **p < 0.01 based on Student’s t test. **(B)** CDC27 expression was detected in normal dorsal ganglia cells and 5 NB cell lines by western blot. GAPDH was used as a reference control.

Supplementary Figure 2
**(A, B)** Knockdown or overexpression transfection efficiency was validated by q-PCR and western blot.

Supplementary Figure 3 The CDC27/ODC1 axis accelerated the wound healing ability of the indicated NB cells. Representative images of wound healing assays are shown. The means ± SD of triplicate samples are shown. ***p < 0.001 based on Student’s t test.

Supplementary Figure 4 Knockdown of CDC27 inhibited ODC1 expression **(A, B)**, and the CDC27/ODC1 axis promoted proliferation and metastasis in SK-N-BE(2) cells. Representative images of colony formation **(C)**, Transwell **(D)**, and wound healing assays **(E)** are shown. The means ± SD of triplicate samples are shown. *p < 0.05, **p < 0.01, ***p < 0.001 based on Student’s t test.

Supplementary Figure 5 GSH assays **(A, B)** and iron assays **(C, D)** were performed in the indicated treated cells.

Supplementary Figure 6 The indicated cells were treated with BSO, WST assays were performed **(A, B)**, and GSH levels **(C)** and MDA contents **(D)** were detected. Means ± SD of triplicate samples are shown. **p < 0.01, ***p < 0.01 based on Student’s t test.

Supplementary Figure 7
**(A, B)** The expression of indicated ferroptosis-associated markers was detected by western blot.
