# Supplementary figures and images for: CDC27-ODC1 Axis Promotes Metastasis, Accelerates Ferroptosis and Predicts Poor Prognosis in Neuroblastoma
Source: Front Oncol. 2022 Feb 15;12:774458. doi: 10.3389/fonc.2022.774458 (PMC8886130; doi:10.3389/fonc.2022.774458)

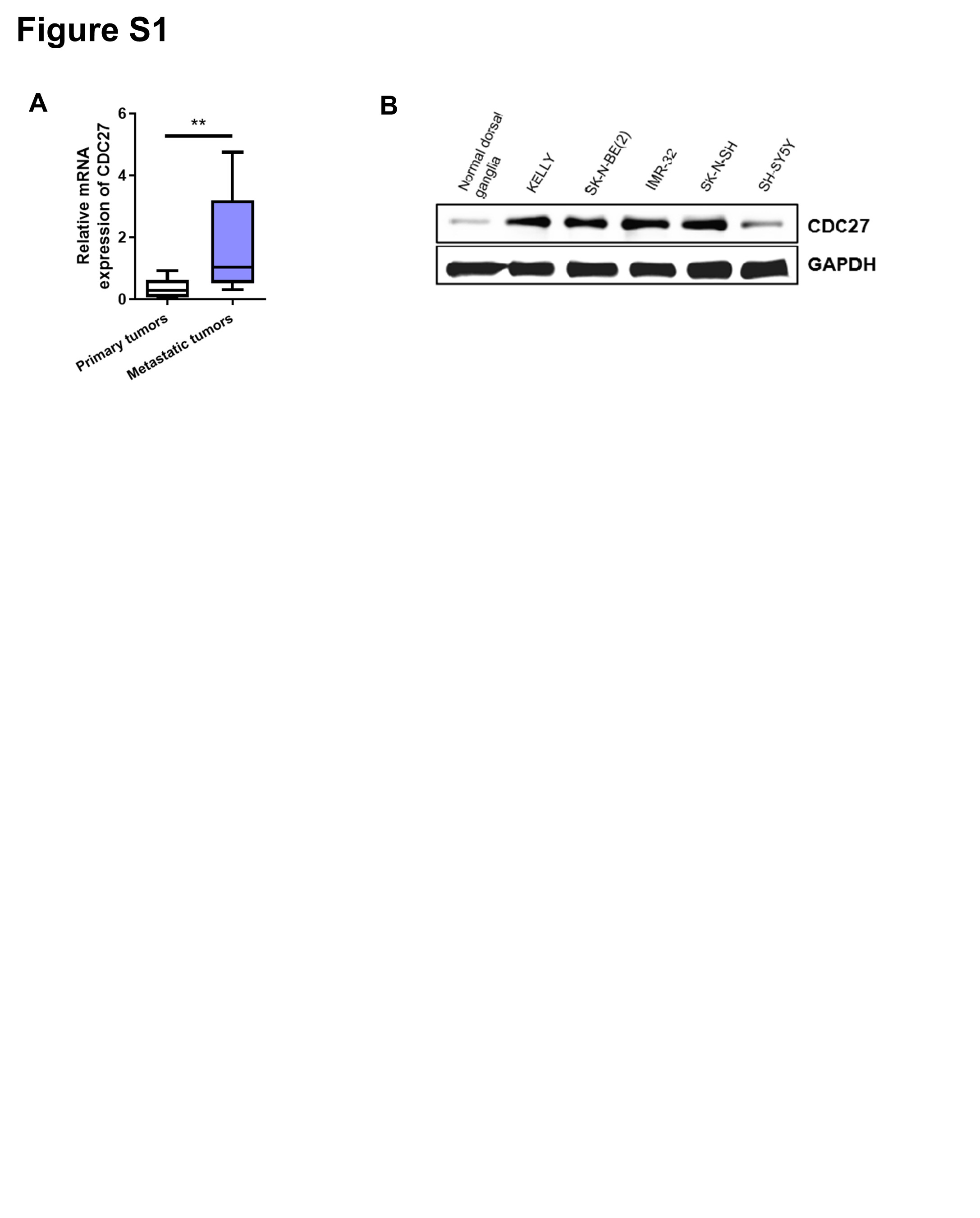

Supplement: Supplementary Figure 1 — (A) Analysis of CDC27 mRNA expression in 12 pairs of primary and metastatic patient tumor tissues. **p < 0.01 based on Student’s t test. (B) CDC27 expression was detected in normal dorsal ganglia cells and 5 NB cell lines by western blot. GAPDH was used as a reference control. [file Image_1.tif]

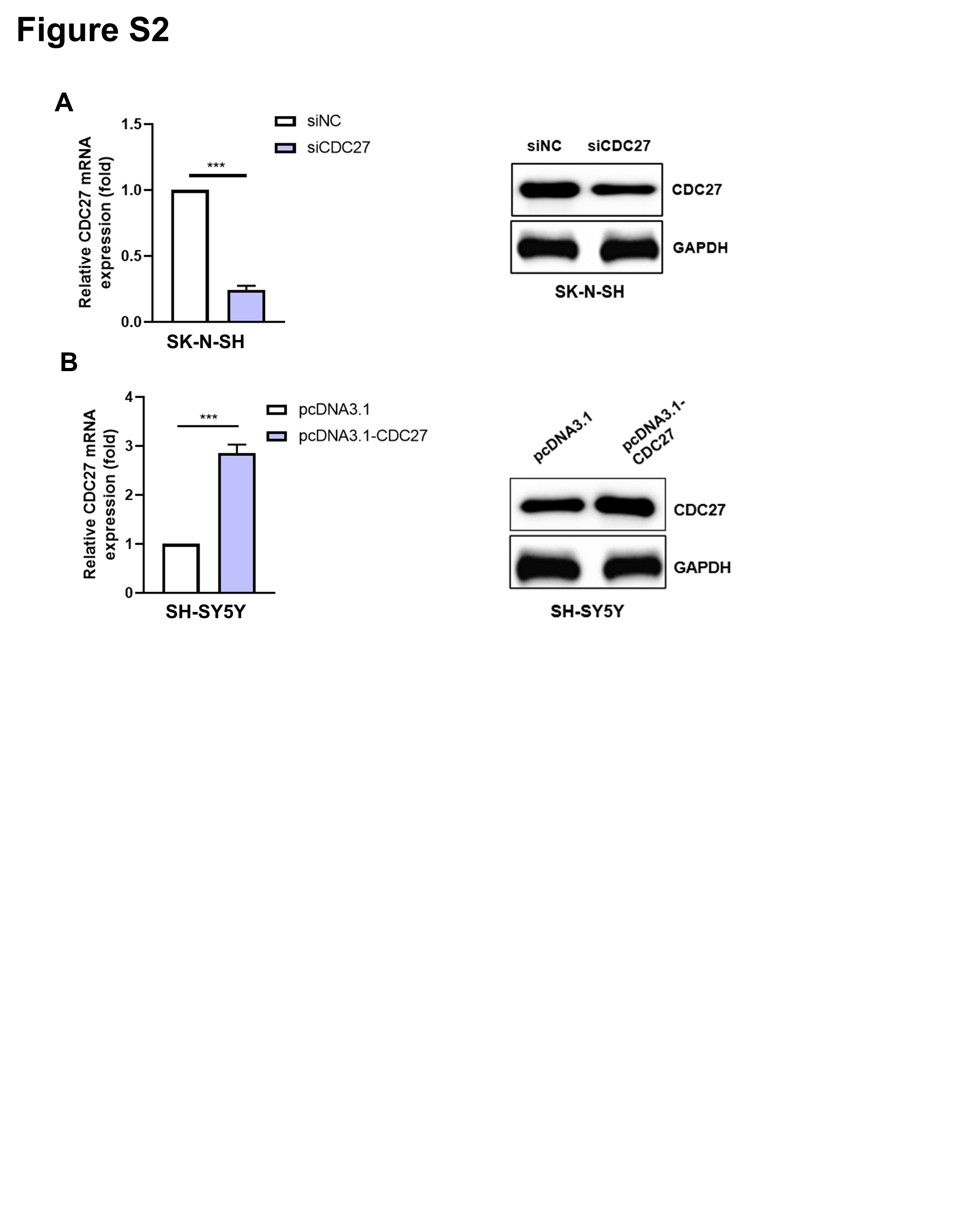

Supplement: Supplementary Figure 2 — (A, B) Knockdown or overexpression transfection efficiency was validated by q-PCR and western blot. [file Image_2.tif]

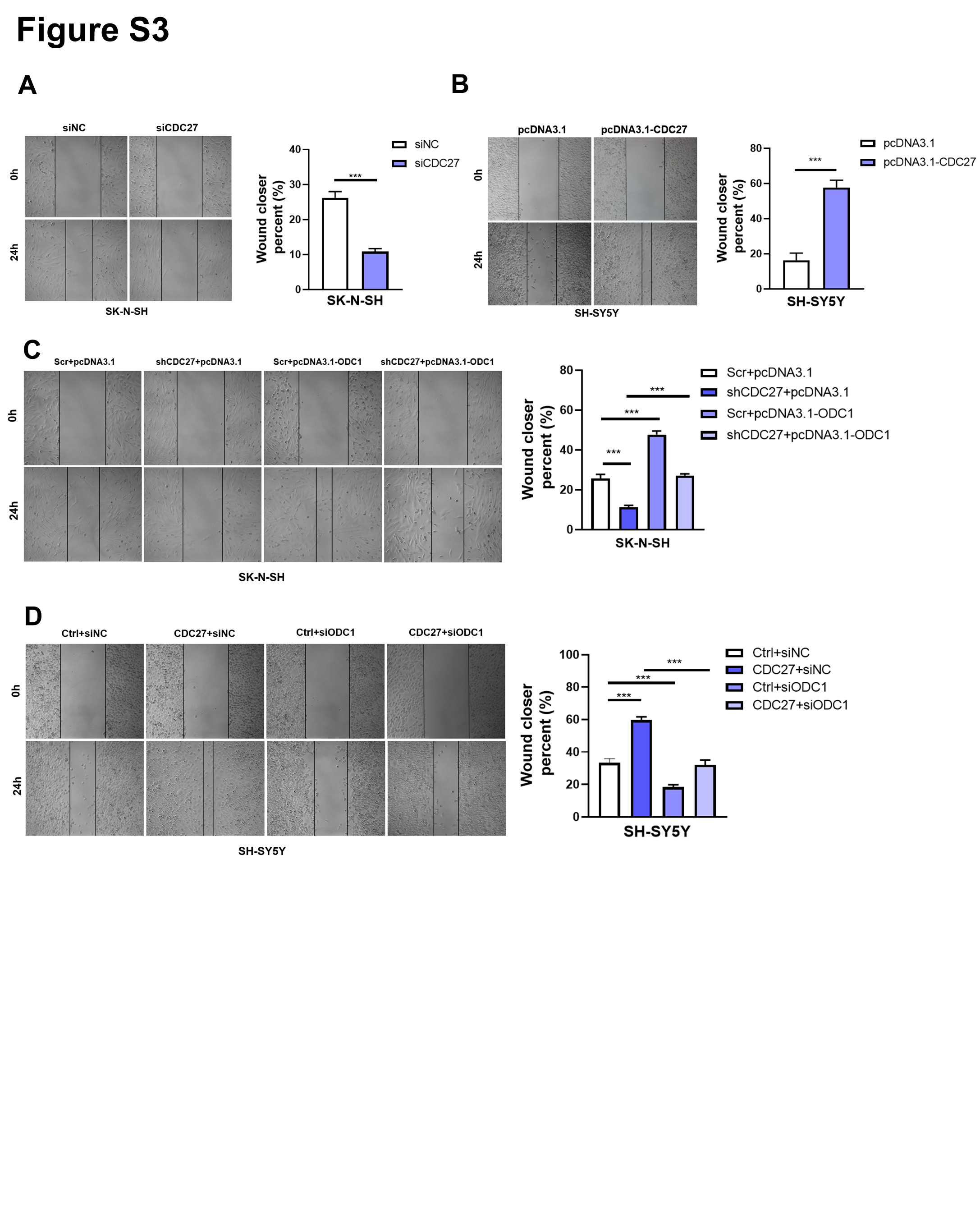

Supplement: Supplementary Figure 3 — The CDC27/ODC1 axis accelerated the wound healing ability of the indicated NB cells. Representative images of wound healing assays are shown. The means ± SD of triplicate samples are shown. ***p < 0.001 based on Student’s t test. [file Image_3.tif]

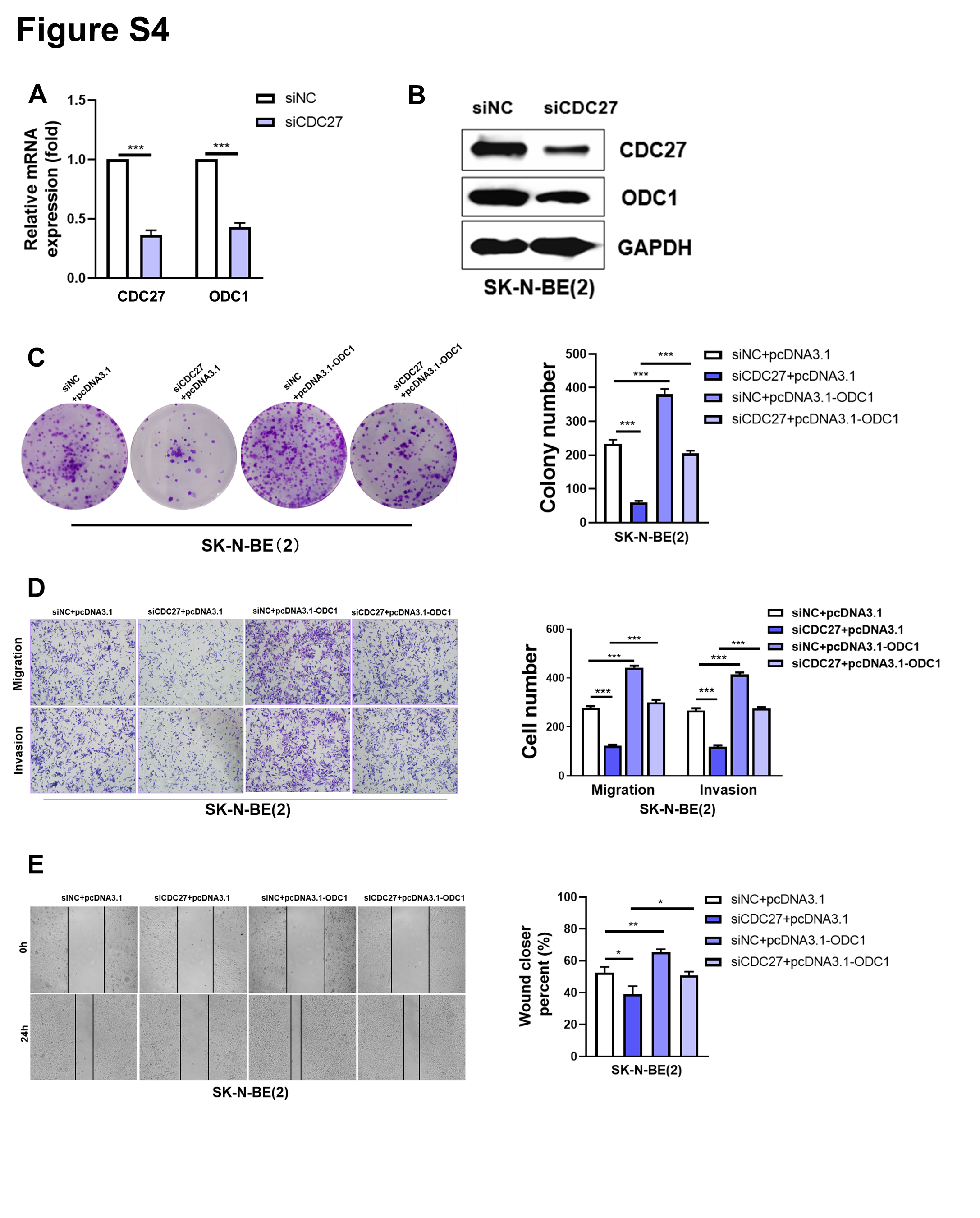

Supplement: Supplementary Figure 4 — Knockdown of CDC27 inhibited ODC1 expression (A, B), and the CDC27/ODC1 axis promoted proliferation and metastasis in SK-N-BE(2) cells. Representative images of colony formation (C), Transwell (D), and wound healing assays (E) are shown. The means ± SD of triplicate samples are shown. *p < 0.05, **p < 0.01, ***p < 0.001 based on Student’s t test. [file Image_4.tif]

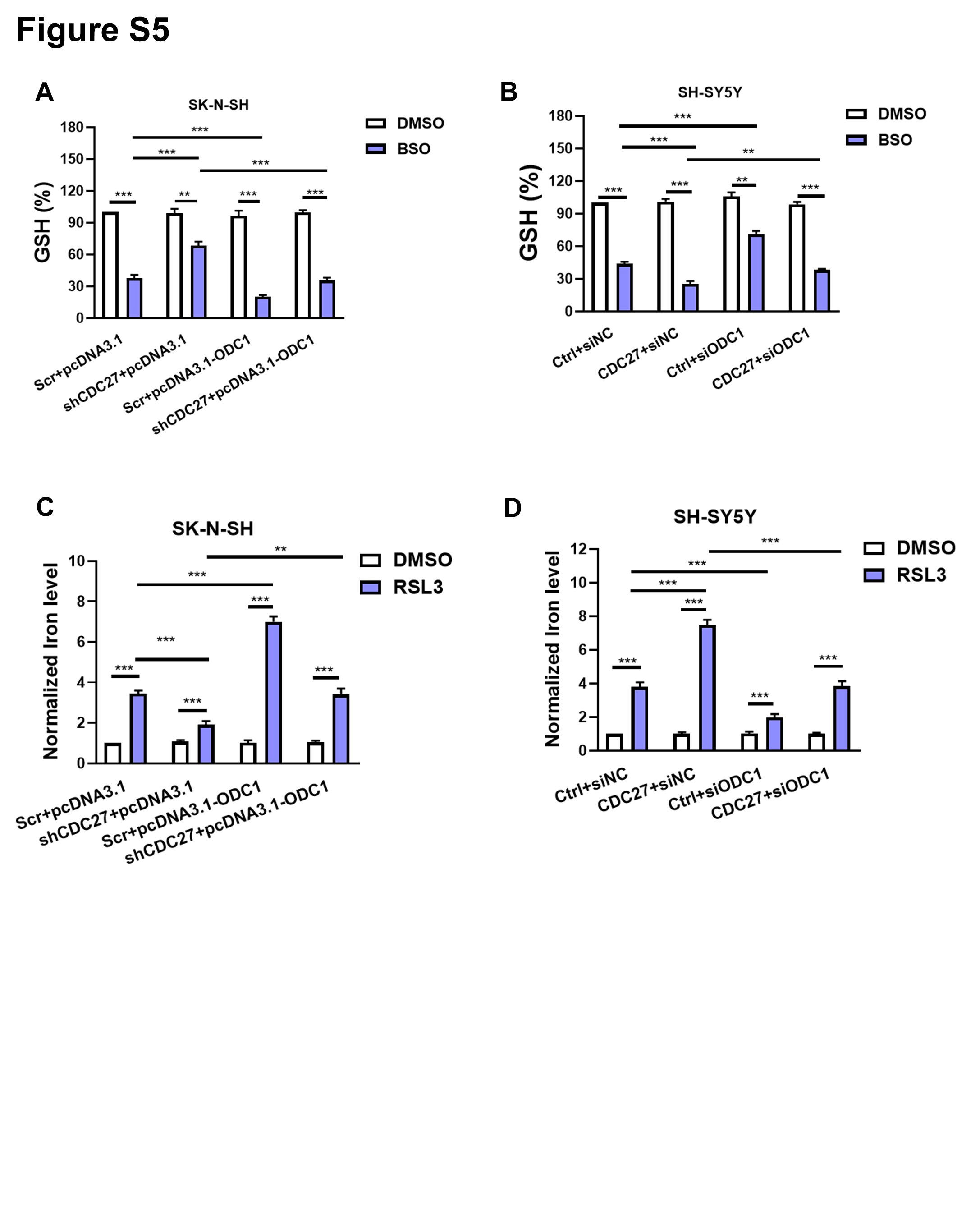

Supplement: Supplementary Figure 5 — GSH assays (A, B) and iron assays (C, D) were performed in the indicated treated cells. [file Image_5.tif]

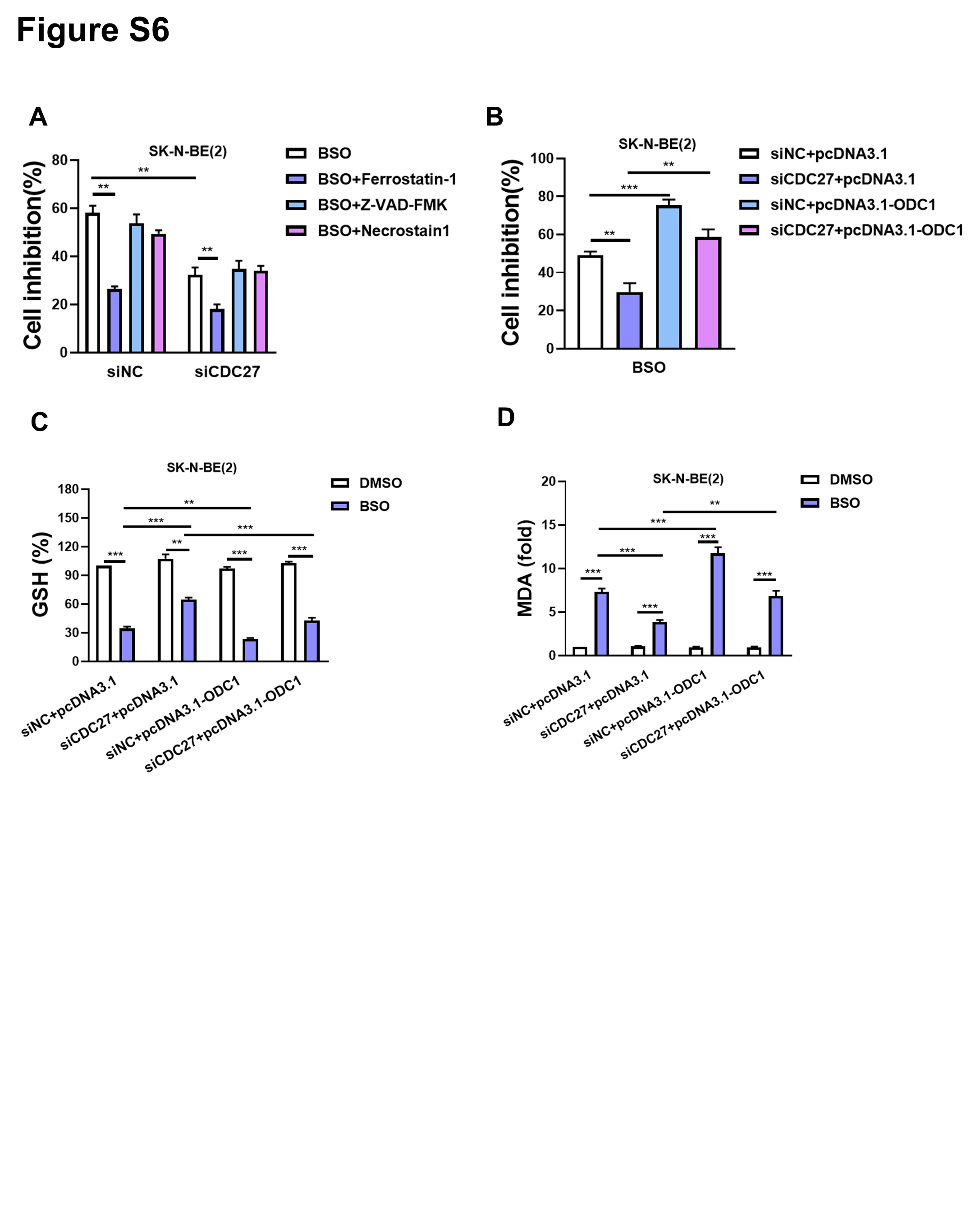

Supplement: Supplementary Figure 6 — The indicated cells were treated with BSO, WST assays were performed (A, B), and GSH levels (C) and MDA contents (D) were detected. Means ± SD of triplicate samples are shown. **p < 0.01, ***p < 0.01 based on Student’s t test. [file Image_6.tif]

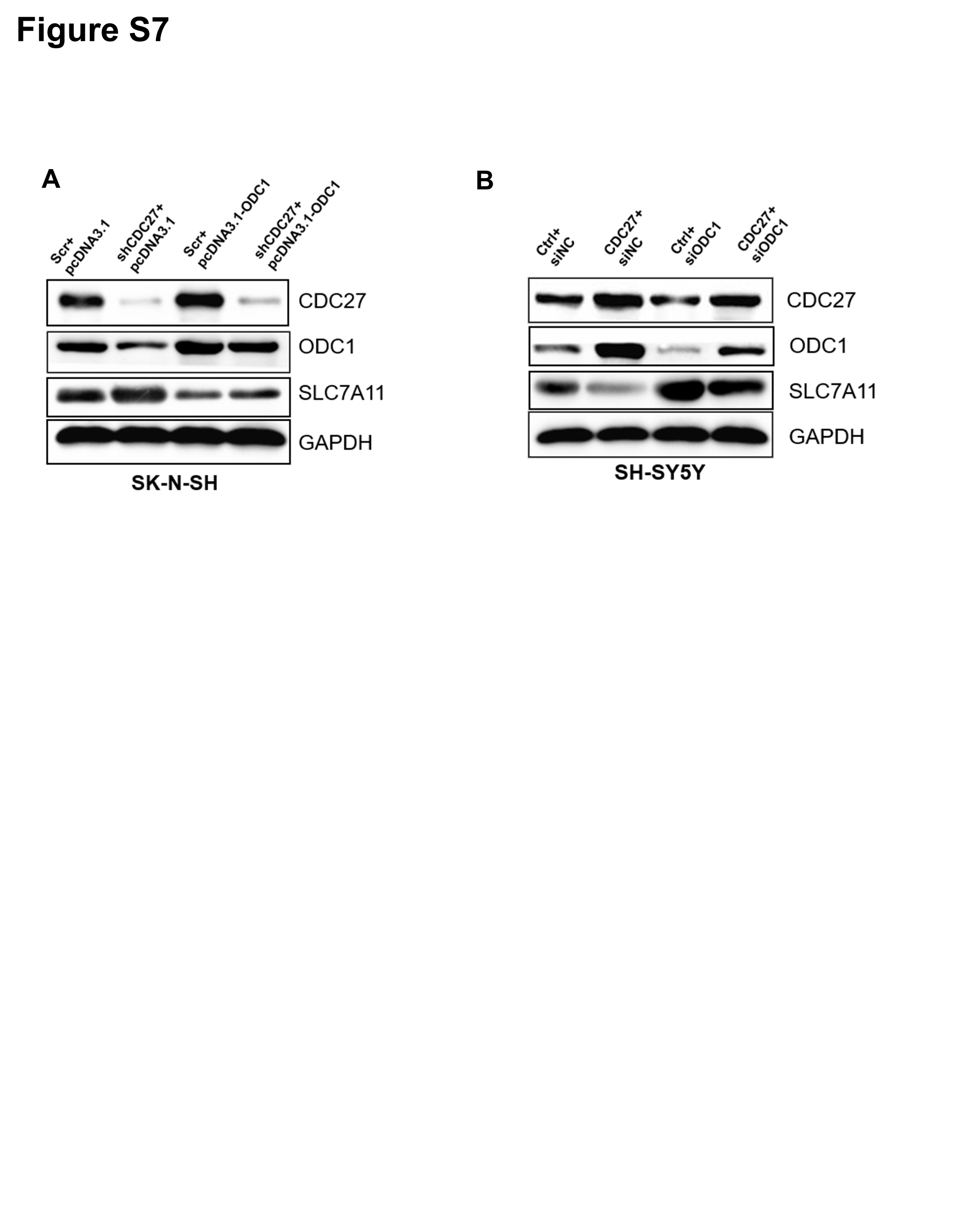

Supplement: Supplementary Figure 7 — (A, B) The expression of indicated ferroptosis-associated markers was detected by western blot. [file Image_7.tif]

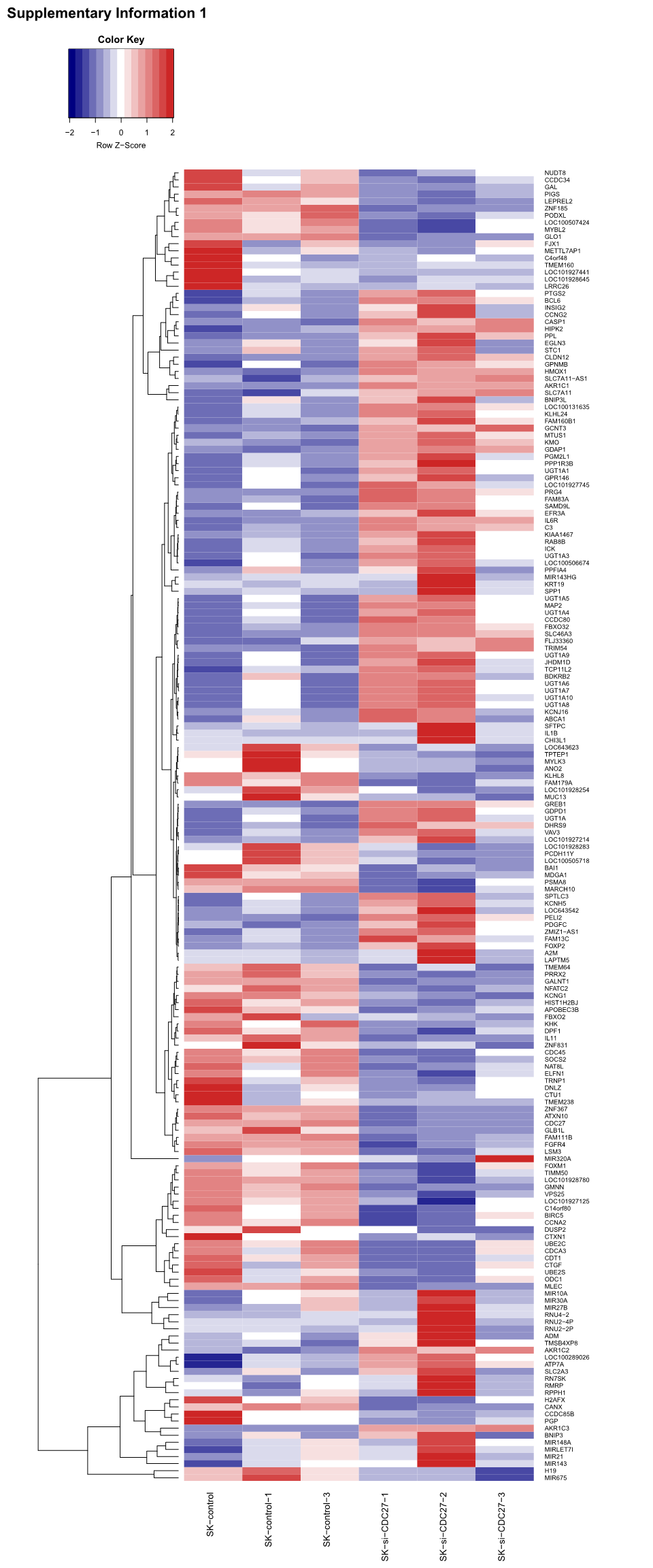

Supplement: Supplementary file 8 [file DataSheet_1.zip › Supplementary Information 1.png]
