# Supplementary material for: CDC27-ODC1 Axis Promotes Metastasis, Accelerates Ferroptosis and Predicts Poor Prognosis in Neuroblastoma
Source: Front Oncol. 2022 Feb 15;12:774458. doi: 10.3389/fonc.2022.774458 (PMC8886130; doi:10.3389/fonc.2022.774458)
Supplement: Supplementary file 9 [file Table_1.doc]

**Supplementary Table S1**

| **Characteristic** | **Number of patients**  **NB (n=44)** | **Number of patients**  **GNB (n=26)** |
| --- | --- | --- |
| **Age** |  |  |
| < 18 months  ≥18 months | 31  13 | 4  22 |
| **Gender** |  |  |
| Male  Female  **INSS stage**  I  II  III  IV | 27  17  21  8  5  10 | 12  14  9  8  4  5 |
