# Supplementary material for: CDC27-ODC1 Axis Promotes Metastasis, Accelerates Ferroptosis and Predicts Poor Prognosis in Neuroblastoma
Source: Front Oncol. 2022 Feb 15;12:774458. doi: 10.3389/fonc.2022.774458 (PMC8886130; doi:10.3389/fonc.2022.774458)
Supplement: Supplementary file 10 [file Table_2.docx]

**Supplementary Table S2**

**Primer sequences for qRT–PCR.**

| **Gene** | | **Sense primer** | **Antisense primer** |
| --- | --- | --- | --- |
| **CDC27** | ACTAAACCACTATGCTTACCGAGATG | | CACTCCACCAGATAAGATTTGTTCC |
| **ODC1** | TTTACTGCCAAGGACATTCTGG | | GGAGAGCTTTTAACCACCTCAG |
| **GPNMB** | AAGATTGCCACTTGATGCCG | | TCCCTCATGTAAGCAGAAGGTC |
| **KCNG1** | ATGACCCTCTTACCGGGAGAC | | TGATGCCGCCTACGTTGATG |
| **ATP7A** | TGACCCTAAACTACAGACTCCAA | | CGCCGTAACAGTCAGAAACAA |
| **CDC45** | TTCGTGTCCGATTTCCGCAAA | | TGGAACCAGCGTATATTGCAC |
| **GMNN** | GCCCTGGGGTTATTGTCCC | | AGCGCCTTTCTCCGTTTTTCT |
| **GCNT3** | TCTGGGCTGCTATATGCTGC | | GTTGATAGACCTCTTTGCTGGAA |
| **AKR1C1** | CCTAAAAGTAAAGCTTTAGAGGCCACC | | GAAAATGAATAAGGTAGAGGTCAACATAAT |
| **SLC7A11** | GCTAATTAAAGGTCAAACGCAG | | CCAGCATATGCATACATTCCA |
| **β-actin** | TGGCACCCAGCACAATGAA | | CTAAGTCATAGTCCGCCTAGAAGCA |
