# Supplementary material for: CDC27-ODC1 Axis Promotes Metastasis, Accelerates Ferroptosis and Predicts Poor Prognosis in Neuroblastoma
Source: Front Oncol. 2022 Feb 15;12:774458. doi: 10.3389/fonc.2022.774458 (PMC8886130; doi:10.3389/fonc.2022.774458)
Supplement: Supplementary file 11 [file Table_3.docx]

**Figure S1**

**(A)** Analysis of CDC27 mRNA expression in 12 pairs of primary and metastatic patient tumor tissues. ** p<0.01 based on Student’s t test. **(B)** CDC27 expression was detected in normal dorsal ganglia cells and 5 NB cell lines by western blot. GAPDH was used as a reference control.

**Figure S2**

**(A-B)** Knockdown or overexpression transfection efficiency was validated by q-PCR and western blot.

**Figure S3**

The CDC27/ODC1 axis accelerated the wound healing ability of the indicated NB cells. Representative images of wound healing assays are shown. The means ± SD of triplicate samples are shown. *** p<0.001 based on Student’s t test.

**Figure S4**

Knockdown of CDC27 inhibited ODC1 expression **(A-B)**, and the CDC27/ODC1 axis promoted proliferation and metastasis in SK-N-BE(2) cells. Representative images of colony formation **(C)**, Transwell **(D)**, and wound healing assays **(E)** are shown. The means ± SD of triplicate samples are shown. * p<0.05, ** p<0.01, *** p<0.001 based on Student’s t test.

**Figure S5**

GSH assays **(A-B)** and iron assays **(C-D)** were performed in the indicated treated cells.

**Figure S6**

The indicated cells were treated with BSO, WST assays were performed **(A-B)**, and GSH levels **(C)** and MDA contents **(D)** were detected. Means ± SD of triplicate samples are shown. ** p<0.01, *** p<0.01 based on Student’s t test.

**Figure S7**

**(A-B)** The expression of indicated ferroptosis-associated markers was detected by western blot.
